# Empathy under prolonged stress situation (COVID-19 pandemic)

**DOI:** 10.1192/j.eurpsy.2025.1266

**Published:** 2025-08-26

**Authors:** T. I. Medvedeva, O. M. Boyko, S. N. Enikolopov, O. Y. Vorontsova

**Affiliations:** 1 Clinical psychology, Mental Health Research Center, Moscow, Russian Federation

## Abstract

**Introduction:**

Empathy is a fundamental component of the socio-emotional experience. Emotional empathy describes the emotional reaction of an observer to the emotional state of another person - “emotional contagion”. People with high empathy can become objects of “emotional contagion” in a stressful situation.

**Objectives:**

**The aim** of the study was to evaluate the mutual influence of stress and emotional aspects of empathy.

**Methods:**

Online survey was used. The level of empathy was assessed by “empathy” of I7 (H.Eysenck), stress was assessed by SCL-90R; COPE and CTI (S.Epstein), questions whether they had ever sought psychiatric help, the presence of suicidal ideas and thoughts of death were used. N=157 (139 women) mean age 41,1. 47 had previously sought psychiatric help. Subgroups of “low” and “high empathy” were analyzed.

**Results:**

People who had previously sought psychiatric help did not differ from “healthy” people in terms of empathy.

**Table:** 

	Low empathy (N=86)	High empathy (N=71)	Sig.
GSI (SCL)	0,46±0,39	0,67±0,53	,004
PDSI (SCL)	1,38±0,45	1,53±0,53	,058
PSI (SCL)	26,34±16,29	35,49±18,06	,001
Global Constructive Thinking (CTI)	98,76±13,84	92,83±12,88	,009
Emotional Coping (CTI)	91,32±23,02	82,75±20,79	,022
Focus on and Venting of Emotions (COPE)	9,38±3,04	10,80±2,72	,003
Seeking Social Support for Emotional Reasons (COPE)	9,56±3,37	10,70±3,24	,032
Alcohol/Drug Use scale (COPE)	6,13±2,70	7,15±3,39	,036

Analysis of variance showed: “high empathy” subgroup had a higher level of experienced stress, including higher anxiety, depressive symptoms, and interpersonal sensitivity. Differences in coping strategies and thinking patterns highlight the personality traits of people with high empathy, which affect the stress resistance. The combination of focusing on one’s own emotions and reducing emotional coping does not allow to regulate emotional state under stress. Separately, using correlation analysis, the relationship between the level of empathy and the characteristics of emotional regulation and possible suicidal ideas and thoughts about death was assessed in the subgroup of subjects who had previously sought psychiatric help and in the “healthy” subgroup. Both in the group of those who sought help from a psychiatrist and in the “healthy” subgroup, a connection was found between empathy and the level of emotional regulation. In those who had previously sought help from a psychiatrist, the level of empathy positively correlated with the presence of “thoughts about death”.

**Conclusions:**

Subjects with “high empathy” are distinguished by a higher level of experienced stress, which may be associated with a reduced ability to emotionally cope. Particular attention should be paid to people who have previously sought psychiatric help. Under stressful conditions with high “emotional contagion” they think about death more often and the risk of suicide may arise.

**Disclosure of Interest:**

None Declared

